# Perceived facilitators and barriers of enrolment, participation and adherence to a family based structured lifestyle modification interventions in Kerala, India: A qualitative study

**DOI:** 10.12688/wellcomeopenres.15415.2

**Published:** 2019-11-27

**Authors:** Linju M. Joseph, T. R. Lekha, Dona Boban, Prinu Jose, Panniyammakal Jeemon

**Affiliations:** 1 Centre for Chronic Disease Control, New Delhi, New Delhi, 110016, India; 2 Sree Chitra Tirunal Institute for Medical Sciences and Technology, Trivandrum, Kerala, 695011, India

**Keywords:** Cardiovascular disease, structured lifestyle intervention, community health workers, family based interventions

## Abstract

**Background:** The objective of the study was to describe participants’ and providers’ perspectives of barriers and facilitators of enrolment, participation and adherence to a structured lifestyle modification (SLM) interventions as part of the PROLIFIC trial in Kerala, India.

**Methods:** Family members who had been enrolled for 12-months or more in a family-based cardiovascular risk reduction intervention study (PROLIFIC Trial) were purposively sampled and interviewed using a semi-structured guide. The non-physician health workers (NPHWs) delivering the intervention were also interviewed or included in focus groups (FGDs). Thematic analysis was used for data analysis.

**Results: **In total, 56 in-depth interviews and three FGDs were conducted. The descriptive themes emerged were categorised as (a) motivation for enrolment and engagement in the SLM interventions, (b) facilitators of adherence, and (c) reasons for non-adherence. A prior knowledge of familial cardiovascular risk, preventive nature of the programme, and a reputed organisation conducting the intervention study were appealing to the participants. Simple suggestions of healthier alternatives based on existing dietary practices, involvement of the whole family, and the free annual blood tests amplified the adherence. Participants highlighted regular monitoring of risk factors and provision of home-based care by NPHWs as facilitators for adherence. Furthermore, external motivation by NPHWs in setting and tracking short terms goals were perceived as enablers of adherence. Nonetheless, home makers expressed difficulty in dealing with varied food choices of family members. Young adults in the programme noted that dietary changes were affected by eating out as they wanted to fit in with peers.

**Conclusions: **The findings suggest that a family-based, trained healthcare worker led SLM interventions are desirable and feasible in Kerala. Increasing the number of visits by NPHWs, regular monitoring and tracking of lifestyle goals, and targeting young adults and children for dietary changes may further improve adherence to SLM interventions.

## Introduction

Cardiovascular disease (CVD) is the foremost cause of mortality and morbidity, in India ^1^. Nearly, one of three deaths in India are attributable to CVD ^2^. Within CVD in India, more than 80% of deaths are due to ischaemic heart disease (IHD) and stroke. Although there is huge variation in IHD burden across different states, it remains as the leading cause of death in all states in India ^2^. Additionally, Indians are more likely to be diagnosed with IHD in their most productive life years (mostly below 65 years) than their western counterparts ^3^. This poses additional stress on the health system, as well as on individual and family life and finances.

The Programme of Lifestyle Intervention in Families for Cardiovascular Risk Reduction (PROLIFIC) trial ^4^ sought to deliver targeted preventive care to high-risk families with an index case of IHD through an integrated care model in Kerala. The burden of IHD in Kerala is highest among all states in India with an average 10-year risk of 20% for development of a fatal or nonfatal CVD event among adults ^5^. A positive family history of IHD is a known cause of subsequent cardiovascular event ^6^. Although the PROLIFIC trial strategy is a targeted and high-risk approach, positive family history of IHD is widely prevalent in approximately 20% of families in Kerala. Therefore, the PROLIFIC interventions are relevant to a sizeable population in Kerala. The integrated model in the PROLIFIC trial included active screening for cardiovascular risk factors by non-physician health care workers (NPHW), followed by delivery of structured lifestyle modification (SLM) interventions with appropriate provisions for linkage to primary healthcare services in eligible participants and active encouragement for intervention adherence. The underlying assumption for the trial is the mutual interdependence of the family as a whole system in decision making and behavioural modifications. A life-threatening event in one of the family members in the form of CHD therefore can act as a reason for change of health behaviours in the family. If the family as a whole system supports the change with additional support from the external environment (society), then it may lead to a change in behaviour ^4^.

Acceptability is a necessary but not sufficient condition for effectiveness of an intervention. Successful implementation depends on the acceptability and adherence of intervention, to both intervention deliverers (e.g. healthcare workers) and recipients (e.g. patients or family) ^7^. Family and friends heavily influence dietary habits. For example, better adherence to lifestyle changes are achieved by active support from the whole family ^8^. Lack of knowledge and understanding of CVD in the community pose challenge to treatment adherence ^9^. Additionally, physicians in India perceive that the adoption of dietary modifications in general is very difficult among patients ^10^. Hence, it is very important to understand how and what kind of lifestyle modifications are feasible for patients and their families ^11^.

Based on previous systematic reviews and studies among Indians living in high-income countries, barriers for lifestyle changes are predominantly related to lack of knowledge and misunderstanding of CVD risk factors ^12– 14^. Additionally, interventions have not been well accepted due to a lack of cultural adaptation of lifestyle messages ^15^.

Trials with complex public health interventions often use qualitative evaluation to understand the participant’s view of the intervention and how the various components influenced the intervention ^16^. In addition, qualitative evaluations during the implementation stage of complex interventions may help to inform the potential for scaling up such strategies in similar settings. Substantial qualitative data are available from studies in high-income countries regarding lifestyle changes in people with CVD ^14, 17, 18^. However, there are not enough insights on possible barriers and facilitators of lifestyle changes among those at high risk for CVD in India. Based on previous work conducted in Kerala, identifying key strategies to improve participants’ engagement and programme adherence are important for initiating lifestyle changes in high risk individuals ^19, 20^. Given the lack of India-focused data, we aimed to explore the views of programme participants, their family members and community health workers on acceptability of the PROLIFIC trial interventions. Additionally, we aimed to identify the perceived facilitators and barriers of enrolment, participation, engagement and adherence to SLM intervention in Kerala, India.

## Methods

### Study design

We conducted a cross-sectional qualitative study to understand the experiences of participating in the PROLIFIC trial intervention after families had completed one-year of SLM intervention. It was conducted as part of the ongoing evaluation of acceptability in terms of ‘reach’ and ‘fidelity’ of the PROLIFIC trial interventions. The methods for the PROLIFIC ^4^ trial, including participant inclusion and exclusion criteria and the nature of the randomisation procedure have been published previously. Briefly, the PROLIFIC trial ^4^ is a cluster randomized controlled trial (c-RCT) that aimed to assess the effectiveness of integrated risk management strategy along with SLM for cardiovascular risk reduction in high-risk families. The participants in the trial were adults with family history of premature IHD. Trained NPHWs visited the participants’ families to promote SLM intervention strategies at least once in every two months during the intervention phase. Accredited social health activists (ASHAs), who provide a range of services, including those specific to reproductive, maternal, neonatal, child and adolescent health, acted as NPHWs in the PROLIFIC trial. Of the 750 families enrolled in the PROLIFIC trial, 368 were randomised to the SLM arm. The SLM interventions and care-coordination were facilitated by 120 trained ASHAs. Initially, ASHAs received two-days of training in communication strategies, identifying risk factors, imparting lifestyle education and promoting adherence to medication and lifestyle changes. Subsequent periodic refresher training was given every 3–6 months, which incorporated suggestions from ASHAs and included measurements of both blood pressure (BP) and capillary blood glucose.

### Study participants, recruitment, and sampling

The qualitative study participants represented different stakeholders in the SLM intervention. Firstly, the intervention family members who were participants in the trial were included. We included a range of individuals to ensure representation of both genders, older and younger family members, employed individuals and home-makers. Secondly, we included other family members who were not trial participants but beneficiaries of the family-based intervention and available for the interviews. Finally, the ASHA workers who delivered the SLM intervention and facilitated the care coordination were included in the qualitative study.

We used participants from the PROLIFIC trial intervention arm to sample the study population purposively ^21^. Interviews were conducted in intervention families and among ASHAs who delivered the SLM interventions. The families were chosen with pre-specified criteria; they were required to be in the intervention arm and have been more than 12 months into the trial. The family member with history of IHD was the index case and all adult members of family were eligible participants in the main trial. However, some eligible members could not participate in the study as they were working or studying in a different city during the intervention period. We included non-participants of PROLIFIC study from the recruited families to understand their experiences of the changes within the family brought about by the SLM interventions.

### Data collection

After collecting the first annual follow-up data for the PROLIFIC trial, the research nurses invited the participants to take part in the qualitative study. They provided the participants with an information sheet and noted the family’s willingness to participate. Research nurses then contacted the interested families for further home-based or telephone interviews; telephone contacts were attempted up to a maximum of three times and at different times of day. To assess the response rate to interview invitations, we maintained a detailed record of all participants who were contacted (i.e. all those who agreed, who refused and who could not be reached). Home-based interviews were planned such that participants and other family members (the IHD affected family member and one or two family members who were not part of the PROLIFIC study) were available for interviews. Research nurses invited the ASHAs for focus group discussions (FGDs) and interviews. All FGDs were conducted in conveniently located pre-booked meeting rooms in our institute or in a conference hall, which were private and quiet. ASHAs were provided with travel allowance for attending the FGDs.

We developed topic guides for the semi-structured in-depth interviews and FGDs (available as *Extended data*). The topic guide was developed based on previous literature ^22, 23^ and was translated to the local language (Malayalam). The guide covered participant and ASHA experiences of being in the intervention study ( Box 1), feedback on intervention components and intervention delivery. The topic guide was discussed with members of the PROLIFIC research team who were not part of the qualitative study and amended to ensure it was culturally and contextually appropriate. Three independent female researchers (LJ, DB and LTR) conducted all the semi-structured interviews and FGDs. The FGDs had additional trained note takers. LJ and DB were public health researchers trained in qualitative research methodology who were not related to the trial. LTR was a post-doctoral researcher in sociology with qualitative research experience who was involved in managing the trial but did not have substantial prior interaction with participants. The duration of interviews and FGDs on average were 30–40 minutes. All interviews were audio recorded using digital recorders and were conducted in Malayalam. Full in-depth interview and FGD guides are available as *Extended data* ^24^. All participants were interviewed once.

Box 1. **Interview guide with participant family members and ASHA workers**

**Participants (Family members)**

- Can you tell me about the experience about taking part in the intervention?
- Probe- As an individual
- As a family
- How did you learn about the programme/intervention?
- What did you expect from the programme initially?
- What is the most important thing that made you join the programme?
- What was the easiest part of the intervention to adopt in your daily life?
- Probe-Why
- What was the hardest part of the intervention to adopt in your daily life?
- Probe-Why
- Would you recommend this programme to other people with similar problems to your own? and why
- Is there anything you would have liked to change or add about the intervention?
- Is there anything in particular that you think of that you would like to share regarding these issues (the programme)?

**Interview guide – ASHA (female community health worker)**

- How have come to know about this project
- Tell us about the project’s influence on your everyday work
- Tell us about things that have been difficult in the project and why
- Tell us about things that have been easy in the project and why
- Tell us about your experience of being part of the PROLIFIC intervention. Any suggestions or lessons learned
  Probes -experience with home visits, training classes, intervention materials
- If you were told that the project was going to be implemented in all districts in Kerala, what would you think?
- Anything in particular that you have thought of/think of that you would like to share

### Data analysis

We analysed the qualitative data with the aim to identify the perceived barriers and facilitators of the SLM intervention. Thematic analysis as described by Braun and Clarke was used for the analysis, which allows for analytical flexibility and epistemological independence ^25^. Information relating to the identity of participants were removed before the analysis and replaced with pseudonyms. Data analyses followed the six steps as described by Braun and Clarke. It began with the data familiarization phase ( *Step 1*) as the researchers (LJ, DB and LTR) transcribed the data themselves. Another researcher (PJ) checked the transcribed data to ensure accuracy. Weft QDA software and excel was used to organise, code and retrieve qualitative data. The researchers listened to and read the interview transcripts of the first three participant and two community health workers, and then decided on an initial coding structure ( *Step 2*). The whole data set was read and re-read independently by LJ and DB and coded using the coding structure. Throughout the coding process, discussion among researchers (LJ and DB) took place to ensure consistency of the codes and identification of new codes. If there were any differences in coding, consensus was reached after discussion with another qualitative researcher (LTR). The codes were examined and organized into broader themes ( *Step 3*). The themes were compared against the study objectives to ensure that those significantly contributed towards the research question were further pursued. Themes were reviewed with the supporting data ( *Step 4*) and after discussion with the research team, they were fully developed and defined with corresponding sub-themes ( *Step 5).* The transcripts or findings were not returned to the participants for comments.

### Ethical approvals

The study is approved by the institutional review boards of the Public Health Foundation of India and Sree Chitra Tirunal Institute for Medical Sciences and Technology. The study protocol for PROLIFIC is registered with the clinical trial registry clinicaltrials.gov ( NCT02771873). Research nurses of the PROLIFIC study invited the participants to take part in the study and took written informed consent. Additionally, the qualitative researchers who conducted the in-depth interviews obtained verbal consent before starting the interview, recording this consent using voice recorders. Written informed consent was also obtained prior to the initiation of FGDs.

## Results

### Study background

**Figure 1.**
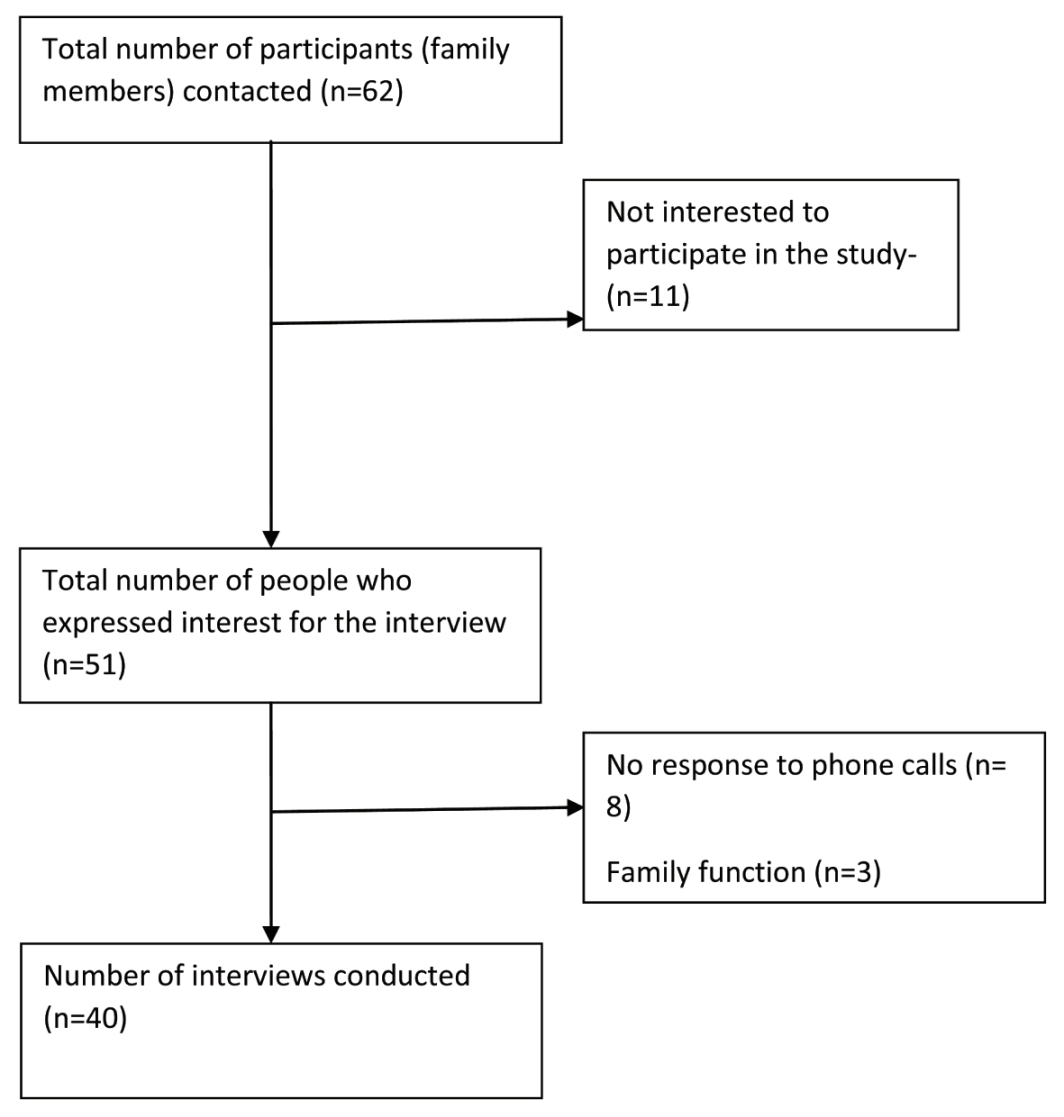
Participant recruitment for interviews.

We contacted 62 participants for the in-depth interviews. Initially, 11 participants refused their participation. Later, three members declined to participate citing inconvenience due to family function. Despite two repeated attempts, eight other participants did not respond to the telephone calls ( Figure 1). Finally, we conducted 40 in-depth interviews with the participants. The participants who refused to participate in the interview were employed and cited job commitments and inconvenience during the timing suggested for the interviews. Additionally, 10 other family members (non-participants in the PROLIFIC trial) were also interviewed. Thus, we conducted in-depth interviews with 50 individuals.

We conducted three focus group discussions (FGDs) of eight ASHAs each (n=24). Some of the ASHAs experienced difficulties in communicating their perceptions in the FGDs and therefore we conducted additional in-depth interviews with six more ASHAs after the FGDs. De-identified transcripts for each interview and FGD are available as *Underlying data* ^24^.

**Figure 2.**
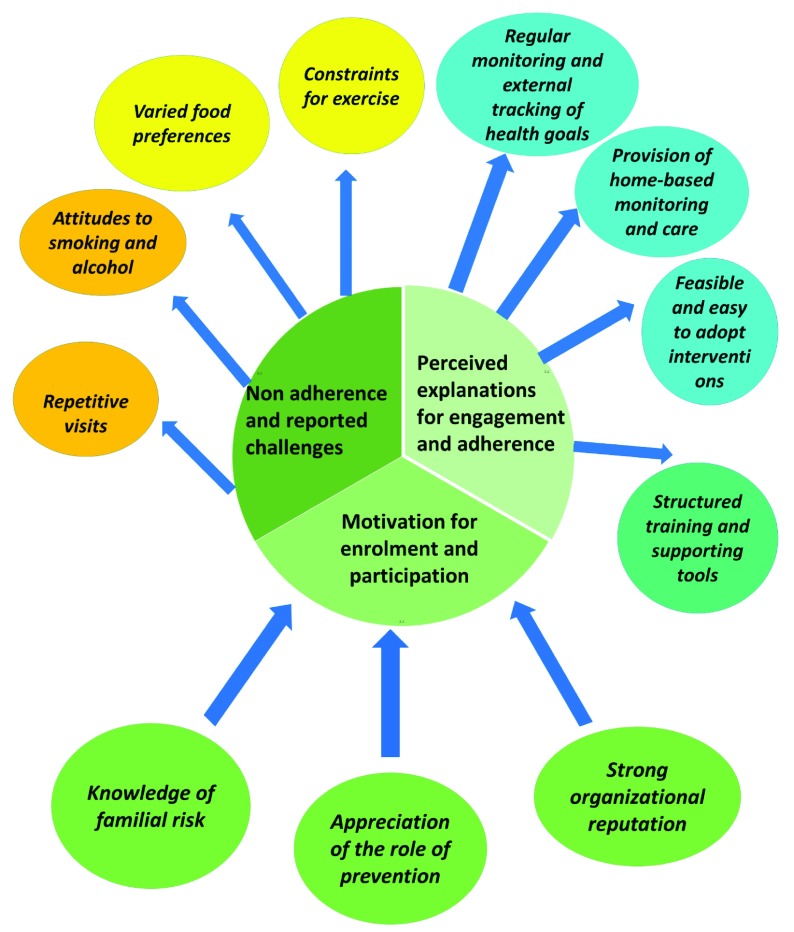
Themes and sub-themes.

The age range of the participants was 19–57 years ( Table 1). The data are presented in three themes and their sub-themes ( Figure 2). First, we presented family’s experience of participating in the trial and generated three themes: ‘motivation for enrolment and participation in the lifestyle intervention’, ‘perceived explanations for engagement and adherence in the SLM interventions’ and ‘reasons for non-adherence and reported challenges’. The sub themes for each theme have been described narratively and summarized in Table 2.

**Table 1.** Demographic characteristics of in-depth interview participants in PROLIFIC intervention group.

Characteristics	Participants in the trial (n=40)	Other family members (n=10)	ASHA workers (n=6)
Age, years (range)	19–57	19–67	41–56
Female, n	31	4	6
Married, n	36	9	6
Education, n			
Less than high school	7	2	0
Completed high school	22	6	3
More than high school	11	2	3
Employment status, n			
Employed	10	4	6
Home maker	23	4	0
Retired	1	0	0
Student	3	2	0
Unemployed	3	0	0

**Table 2.** Themes and illustrative quotations.

Themes	Sub-themes	Illustrative Quotations
**Motivation for** **enrolment and** **participation**	Knowledge of familial risk	• Since my father was ill, we also had risk of getting diseased. Hence, I joined the program (Telephone participant, *TP2,Male,36years)* • I had thought that it would be nice for all of us to get checked. Since my son’s father had such a problem I thought it would be good to know if anyone else had( *P7, Female,44years)* • Since my brother has disease, I had the risk. I had the same disease as my brother years back( *P12,Male,47years)* • Some of my participants were aware of the risk I think. They were already using less amount of oil and avoid caloric rich food. ( *FGD1, ASHA1*).
	Strong organizational reputation	• We feel more confident that there is someone to take care of our health. Especially in the case of Chitra, they will take care and is giving us advice at regular interval and feel that they are helping us *(P13, Female,49years)* • I believe that I had a positive approach from the family due to the name “Sree Chitra”( *FGD2, ASHA1)* • They have respect while hearing that I am coming from SreeChithra Hospital as a part of the program. It is good experience rather than other programme home visit. ( *FGD2,ASHA7*) • After my husband suffered from heart attack, we are already controlling our diet well. A lot of lifestyle changes happened to my husband post the event( *TP1, Female, 56yrs*)
	Appreciation of the role of prevention	• I feel it is good or good in the sense that they will get themselves checked and thus can know about it or prevent it. Only when such programme come will everyone get checked. Or else who will listen to such things as getting checked. *(*Other family member, * OFM4, Male, 46years)* • It is good for knowing whether we have any disease or not.(P3, Female, 53years)
**Perceived** **explanations** **for** **engagement** **and adherence**	Regular monitoring and external tracking of health goals	• Usually I won’t go for checkups. Only because of being in this project, I am going for monthly checkups and yearly checkup. ( *TP13,Female,45years)* • Getting our blood tested for free is a big thing, considering the expense of blood tests.( *TP18, Female,52years)* • The best thing is the blood tests. We may adopt some dietary changes or even some exercise on our own but we would have never got ourselves tested, hadn’t for this programme. Even though we know it is good to get checked but we won’t. We may even think of doing it but will keep postponing it. And the results lets you know where we stand *.(P1,Female,39years)*
	Provision of home- based monitoring and care	• The regular visits by staff and ASHAs in my family, and updating our knowledge on diet control and exercise has helped myself and my family members to make better changes in life. *(TP19, Female,51years)* • We never use to go out to check BP. Now, people are coming to our house to check BP and let us know everything. *(P10,Male,30years)* • All the information the ASHAs gave were known to us but when they come and remind us and also the thought that they are actually coming for us, makes a difference *…(P1,Female,39years)* • Home visits by ASHA is the best part. The instructions and knowledge shared by ASHA during the visit is being followed in the family. Before their visit, we were not that aware of all these things. They will speak about diet control, exercise etc. The instructions given by them are practically feasible and when it is useful for all in the family, it made some changes in the family. *(P4,Male,56years)* • I: You have said that you were already aware about the health issues and other information. What was your motivation to bring out the changes during this time? • R: Monthly visits by ASHA and monthly checkups done for the family were the motivation. *(P20,female,29years)* • Even if I am aware or informed about health, the dedicated staff visits in my family and monthly checkups are the main component that make me bring the changes. *(TP20,Female,54years)* • If someone pressurises, then we will continue to do. But once the programme stops, I think I can adapt the dietary changes.(P15, Male, 21years)
	Feasible and easy to adopt interventions	• My lifestyle changed a lot especially in food control which I would continue till the end of my life.( *P20,female,29years)* • I will walk in the morning for some time. I used to walk even before being part of this project. But was not regular. Now I have started again. I will walk for half hour to 45 minutes. After the visits by ASHA, I became more regular. I used to do yoga before. I will do some yoga some times nowadays *.(P4, Male,56years)* • I ride bicycle to and fro and while climbing steeps need to take deep breaths (laughs). These route is travelled daily 5–6 times. It helps in making good pumping. I am brisk even in my 70’s. I ride same as youth of 30’s does. People tell that I am riding so fast. Earlier I was hesitant to follow these because of time constraint but the health worker motivated me in doing all these.(P16, Male, 68years) • I only use little oil while tempering mustard seeds. I avoid “varathadum (fried) and porichadum(deep-fried)” I give my children some-times fried items such fried fish but will use only little oil. I find it difficult to have fried food due to gastric burn. I like vegetables very much. And I have increased having them. (OFM2, Female, 53years) • I was able to reduce the amount of salt and oil. When they started to check BP and sugar monthly, it became a motivation to reduce the oil and salt.(TP12) • Earlier we used 4 litres of oil as to prepare snacks in the evening…also to fry chicken and fish. But now it got reduced to one a half litres….and sometimes it’s not fully used…(laughs). Also we do not use reheated oil. Once used…we discard it. Earlier it was not so…we take the left over oil for preparing other dishes...(OFM 10, Male, 50years) • Reduction in oil and sugar and salt consumption was easier to follow. My wife puts one small spoon sugar if only I ask for(laughs).(P16, Male, 68years) • I have reduced oil considerably. And that too since the last one year. My cholesterol level too was high in the result. Earlier we used to have fried item but now we have totally avoided such food and doesn’t have the habit of taking food from outside home.( *P14,Female,50years)*
	Structured training and supporting tools	• I think the training and hand book given was very useful and it had pictures so I could tell people better. Not everything but the book was written well and I could use the information well *.(ID, ASHA1)* • The training was done one after the another. They have been helpful in helping me to impart information to families who were struggling with disease. I have learnt a lot from the training especially how to deal with people with some kind of risk factor *..(ID, ASHA4)*
**Non-adherence** **and reported** **challenges**	Constraints for exercise	• I will walk in the morning in house itself. Going out to walk is a problem. Then I do all the household chores in house which is also a good exercise.( *TP1, Female, 56yrs*) • I think that since everything is normal for me, they did not mention about doing exercise. She didn’t tell me regarding walking. I do all household chores at home regularly. That should do as my exercise.(TP3) • I will do a lot of household chores like washing clothes, mopping, going to shop to buy groceries. I do not specifically do any exercise.(TP14) • They have advised us regarding walking. I am walking always. My job requires a lot of walking. So, there is no need to walk extra.( *P6, Female, 51 years)* • Doing exercise daily is not feasible for me because of no time. *(TP10, female,30 years)* • ASHA told me about exercise. I am doing the entire household chores including sweeping and mopping. I won’t have enough time to go out for exercise.( *TP20, Female, 54 years)* • Even if the ASHA workers come and say it is us who will have to do. So I am not sure. I think it mostly we are lazy so if they are coming more times per week and insists then we may do.( *P2, Female, 40 years)* • I was not able to do any exercise. My health conditions doesn’t let me do it. That’s is the main reason. And also when I do all the household chores, am I not walking? So why doesn’t that count as exercise? At least 3 hours in a whole day I will be household chores so isn’t that enough? ( *P2, Female, 40 years)* • I have heavy work in the workshop. No other specific exercises. I use a two wheeler to go for work. *(P11,Male,25years)*
	Varied food preferences	• We don’t use “chamba”(red-unpolished variety) rice because no one likes it in family. So we use the white ponni (polished )rice itself *(TP16, female,52years)* • My son will have meat items from outside. He is not ready to avoid it. My daughter has high cholesterol. Still, she will have the meat items *.(P17,Female,50years)* • Whenever we buy fish, I prepare fish fry for him while we have fish curry. I will prepare meat items for him alone. My son doesn’t prefer vegetables *.(P17,Female,50years)* • I reduced the amount of salt added to the dishes. Following that, parents started to put extra salt in their rice or curry. My mother says that without salt, the food is tasteless. They “NEED” salt in good amounts *.(TP7,Female,35years)*
	Repetitive visits	• Even with prior appointment from the family, the members will be missing and I eventually have to visit two or more times in the same family. ( *FGD1, ASHA6)* • In one family I had to make several visits as the son is an alcoholic and I could not talk to him. ( *FGD2,ASHA1)*
	Attitudes to smoking and alcohol	• The families especially the female members say that the male members have an alcoholic issue but the male members never say that to me, In fact most of them lie regarding alcoholism. ( *FGD2, ASHA4)* • They do say it but then again they can only say it. It is upto us what we do ( *referring to drinking and smoking*( *OFM4, Male,* *46years)* • He was in Gulf before. When he came back here, due to bad friendships…. These bad friendships will catch always rich people. He is addicted to alcohol. We can only tell right?( *ID -ASHA2)* • I have tried to talk about ill effects of smoking but young men do not take any notice. The pamphlets just say about the side effects. I think pictures should be included like the one they show before movie. After that they may be more willing to listen.( *F GD1, ASHA3)*

### Motivation for enrolment and participation

Most participants described their interest for being in the intervention based on their individual circumstances. This was mainly influenced by knowledge of familial risk and the reputation of the organisation implementing the study.

***Knowledge of familial risk.*** Participants reported being aware of the risk associated with family history of IHD. In particular, participants with prior knowledge or an awareness that cardiovascular risk is familial were keen to join the intervention. For example,

*“I am already aware that since my parents have diseases, I am also at risk of developing. There are changes in hormones in my body as well.” (Participant (P)20, female, 29 years)*

However, not everyone had prior knowledge of familial risk. For many, source of awareness appeared to be from the information given when they were contacted for being a part of the programme.

*“I thought that they contacted me because my phone number was given in the hospital.*
*When they came and explained regarding the project, I understood that they contacted me because I am also at risk of having heart diseases.” (P10, Male, 30 years)*

ASHAs also agreed that some families were aware of their risk and some had no awareness of familial risk.

“ *The patient and family members got an idea from the hospital that all his sons and daughters have a risk for having heart disease. I think they were happy that I also told them similar things they heard from the hospital.” (FGD2, ASHA2, Female)*

***Appreciation of the role of prevention.*** Some participants decided to be a part of the intervention owing to the preventive nature. They also emphasized how prevention may have a beneficial effect on their overall health.

*“I felt that there will be tests done and we will get the results, one for us and one with you. Sometimes when we do tests outside, the results take some time to arrive. So I felt that it would be good to know about our health status earlier on, rather after having some problem or may be able to prevent disease.” (P16, female, 52 years)*

However, one participant felt that prevention may not be always possible. The contradicting views were mainly regarding the limits of health benefits that can be achieved from a preventive programme.

*“I don’t feel that doing all these can prevent us from getting disease. There is many other factors not just oil or salt. There is adulteration in much of foods. And if you are poor then there are hardly many things we can afford and then what to avoid. “(P11, Male, 25years)*

Thus, most of the participants who found the programme acceptable had some awareness regarding possible risk and were willing to make some changes to prevent cardiovascular disease.

***Strong institutional reputation.*** Participant’s accounts suggest that the strong reputation of the implementing organisation among the community led to acceptance of the SLM intervention. Participants had good experiences with the organisation when a family member or relative had been treated before at the institution or have heard about the organisation. The trust and reputation made some participants inclined to participate in the programme.

*“The name Sree Chitra was such trustworthy. So I felt that this would be a good programme.“(P12, male, 47 years)*

Similarly, the ASHAs appreciated the acceptance they received from families due to the reputation of the implementing organisation. Being part of a study by a reputed organisation convinced the selected family members to be a part of the programme.

*“The families were very cooperative with me after hearing that I have come as a part of programme run by “Sree Chitra”. This helped me to convey the messages later on with ease. “(FGD1, ASHA 5, Female)*

### Perceived explanations for engagement and adherence

Perceived benefits theme contributed to the understanding of facilitating factors. Within perceived benefits, three sub themes were identified. Monitoring in family, re-assurance when lab values are controlled and easily adoptable changes were the most crucial subthemes in perceived benefits.

***Provision of home-based monitoring and care.*** Most of the participants appreciated regular monitoring of their BP and sugar by the ASHA workers. It helped them in changing their lifestyle to bring the values to optimal level. Indeed, as the following quote illustrates, regular monitoring is beneficial for participants.

*“She will come every month. She will explain everything in detail “just like how doctors explain”. She will take BP, sugar monthly and tell us how to control if it is not normal.” (P19, Female, 58 years)*

Participants appreciated that the health information was given at their home, for their entire family. For example, some respondents perceived that when the lifestyle modification advices provided repeatedly, they acted as a facilitator to the intervention.

*“I am trying my level best to follow the instructions given by them, even though it’s not possible every day. Because of their regular visits, we are able to bring changes in our diet and lifestyle.” (TP (Telephonic participant) 13, female, 45 years)*

Participants emphasized more importance on health information delivered by healthcare workers than the written materials like pamphlets distributed to them.

***Regular monitoring and external tracking of health goals.*** Majority of the participants were eager to have themselves tested and expressed being happy when the lab values or BP are normal. Many participants viewed this as one of the most beneficial aspect of the programme.

*“When we get the yearly result of tests done, and seeing that we are normal is a matter of happiness now.” (TP3, Female, 36 years)*
*“The most positive thing is the annual blood check-up. I am very eager to get the tests and also the results, that gives the sense of knowing that everything is ok or not.” (P2, female, 40 years)*

For many a deviation in the result prompted them to carefully examine dietary habits.

*“When we get ourselves checked like knowing our BP or lab results, we will forced to think why has it gone up and how can I reduce it? I feel like I should control it."(P1, female, 39 years)*

***Feasible and easy to adopt interventions.*** The community health workers imparted the SLM intervention to the whole family. The delivery of lifestyle messages reached all members of the family. However, participants described support from family members were needed to follow the dietary changes. For one participant, his wife’s understanding of the message worked in preparing healthier options and then the change was easier.

*“I won’t eat any fried items. I don’t eat Pappad. I will ask my wife to prepare dishes with very less coconut oil. After the instructions by ASHA, the coconut oil usage is very less. Since my wife also heard from the ASHAs, she was keen to reduce oil usage. Such changes in diet was made to everyone in family, not just for me.” (P4, Male, 56 years)*

Most participants described the desire to follow a healthier diet. Participants, who felt they had made changes, described them mostly in terms of reduction of usage of oil, salt and sugar.

*“For myself, husband and two small children we used around 1.5 kg of oil per month. Now we are able to limit the consumption to within half kg oil per month. We totally avoided frying fish and meat. We will give fried items only to children within this half kg oil limit.”(TP4, female, 49 years)*

ASHA workers also felt that the participants were making some changes, which they could follow.

*“Not following the exact instructions that we gave about diet, but some changes implemented. For example, avoided use of palm oil, reduced salt, maida, fried food etc.” (FGD3, ASHA5, female)*

Many women reported preferences of taste being the deciding factor in cooking meals. However, since they were responsible for cooking, they had more control over the measurements of salt or oil being used for cooking. Hence, they made changes, which they could achieve easily.

*“My husband prefers oil and salt in food. But still we reduced oil and salt. Before we used 2-3 packets of oil. Now we are using only 1.5 kg of oil per month. We avoided fried items. Regarding salt, in place of two spoons, I add only one spoon now.” (TP9, female, 50years)*

Some of them did not feel that they had made any particular changes, as they believed they were already having a balanced diet. On the contrary, few participants described the interventions as useful as it made them aware of their portion sizes.

*“Before I was 70 kg in weight. Now I am weighing 62 kg. I reduced the weight through reducing the amount of food eaten at night, avoiding rice for dinner. If I eat rice, I would eat only less amount” (TP7, Female, 35 years)*

A few participants noted that recording their dietary and physical activity in the diary helped them to be engaged with the interventions. However, some of them struggled to regularly document dietary and physical activity details in the diary.

*“I used to read the information printed in the diary, initially. I will not write diary regularly. Maybe once in two weeks, I may write, most of the time I forget.” (TP2, Male, 36 years)*

All ASHAs reported that the intervention was feasible for them to deliver. The positive factors that enabled them to deliver the intervention were training and tailored materials such as handbook and healthy recipe book for ASHAs.

***Structured training and supporting tools.*** Most of the ASHAs agreed that trainings before and during the programme helped them to understand the lifestyle messages.

*“Staff in the Panchayat area got training. I am in corporation area. I work as ASHA under hospital. So public health nurses used to check all these if there are NCD (non-communicable disease) clinics. After coming here (PROLIFIC study) in the third training, I learned to check BP and sugar, which was very useful.” (IDI, ASHA 4, Female)*

### Non-adherence and reported challenges

Two sub themes identified in reported challenges were relating to constraints to exercise and food preferences among family members.

***Constraints for exercising.*** Respondents described contextual factors, such as lack of time and space constraints for exercise. Employed participants expressed constraints in finding time due to work commitments. On the other hand, homemakers struggled to take out time for themselves due to household chores.

*“I find exercise as a difficult thing. I don’t get enough time to spend for exercise. I am a housewife. My children are studying. So, the entire day, I will be busy with household chores.”(P3, female, 53 years)*

Many female participants perceived that exercise as unnecessary and justified their activity levels as they engaged themselves in household chores. In one case,

*“I am having a lot of household work starting from 4am to 10pm. That itself is a big exercise. I don’t do any other extra exercise.” (TP18, Female, 52 years)*

ASHA workers had similar experiences after family visits. They felt that mostly women considered household chores as enough exercise.

*“Almost all female members say that doing house hold work and kitchen work is itself is a big exercise.” (FGD3, ASHA1)*

Thus, most of the participants did not actively seek help for exercise, as they perceived themselves as having adequate physical activity. Similar views were expressed by ASHAs regarding constraints to exercise. ASHAs reported that participants described lack of time for exercise due to housework or job timings.

***Varied food preferences.*** Most homemakers described that the challenges they faced in making dietary changes were due to varied food preferences among family members. Even though the focus of the intervention was changing habits of families as a whole, it was met with difficulty. They specifically highlighted that they did not make many changes to diet of their children. For example, one participant said,

*“Whenever we buy fish, I prepare fish fry for him while we have fish curry. I will prepare meat items for him alone. My son doesn’t prefer vegetables.” (P17, Female, 50 years)*

While the above stated challenge was due to preference made by individuals in a family, some respondents view financial constraints a reason for the food choices.

*“We are not able to eat fruits everyday as per the given instructions. We can give that only to my husband because of his health priority. Fruits are expensive. So, we are not able to eat it daily.” (P7, Female, 44 years)*

Thus, families struggled to cope when dietary changes affected preferences of children. Often in such situations, they did not comply with the lifestyle instructions.

Some participants expressed their desire to change. However, they had trouble knowing how to begin the lifestyle changes. For example, a participant noted

*“I would like to reduce the amount of food I eat. But I don’t know how to do and where to start”. ASHA will give instructions regarding everything. But I don’t know how to start. I am thinking on how to reduce the food intake.” (TP6, Female, 44 years)*

Some participants responded that they were reluctant to follow the advice received. This was either due to the fact that they had some prior information regarding lifestyle changes when their family member had the cardiovascular incident. Hence, they considered themselves to be following a healthy lifestyle. While some others did not want to change their habits.

*“There is some diet chart given in diary. I haven’t tried to follow that. We were having the usual foods from my childhood. I didn’t want to change from that.” (TP2, Male, 36years)*

***Attitudes towards smoking and alcohol.*** Most of the ASHAs confided that they asked about smoking and alcohol habits in the family. They reported discussing it with the female members to understand the scenario better. The advices were given to the family members regarding the need for quitting smoking. However, they found that the smokers and alcoholics were in denial of the harmful effects.

*“One of the participants was not ready to accept the harms of smoking. He started questioning me if I was sure about not getting cardiac disease if he stopped smoking and drinking.” (FGD3, ASHA1, Female)*

***Repetitive visits.*** The main barrier for the intervention delivery was the difficulty in meeting with every family member. Mostly they would be able to meet with the female members of the family.

*“Some time we arrange a family visit and then when we go and meet the family, not all members will be there…this would mean I will have to go twice or thrice to a family to meet all the members of the family” (FGD1, ASHA 8, Female)*

One of them explained that households where all are employed is difficult to arrange and meet. This would lead to arrangement of visits at difficult timings for both family members and for ASHA workers. She went on to describe a possible reason of the educated families not requiring ASHA visits.

*“…They received all materials from me. But when I check sugar or BP, they tell that ‘This is of no use and I know all these, my doctor had told all about this.’ They both are working and will be available only late hours so my son used to accompany me. They are educated and have a feeling that they have enough awareness.” (IDI, ASHA6, Female)*

## Discussion

We sought to understand the acceptability of a family-based cardiovascular risk reduction intervention in high-risk individuals with family history of premature IHD in Kerala. Further, the qualitative study helped us to describe the facilitators and barriers for enrolment, participation and engagement in the SLM interventions. Overall, the PROLIFIC interventions were perceived as acceptable among families based on their interest and engagement in one or more components of the intervention.

Prior knowledge of risk factors and recognition of the importance of IHD prevention facilitated enrolment and participation. The role of family’s perception of the prior cardiac event is important in shaping the family’s response to the situation as postulated by the ABCX model of family systems. For example, in the ABCX model the event (A), the family’s resources (B), and the family’s perception of the event (C) all play a part in determining the family’s response to an emerging priority or need (X) ^26^. However, our results indicate that awareness of risk alone is not enough for initiating and sustaining lifestyle changes. Even though some participants agreed that they were at higher risk of future IHD, the underestimation of risk may have contributed to the lack of initiation of prevention strategies. Previous study findings confirm similar underestimation of risk perception among patients at high-risk of CVD ^27^. The double ABCX model is primarily a process of adaptation to stressful situations and hence the response to the event may be different after the acute-phase, as the families begin to adapt to the cardiac event of the family member ^28^.

Despite the lack of focus on children in the PROLIFIC interventions, two of the five propositions outlined by Vedanthan *et al.* ^29^ have been found to be true in our study. Firstly, as they postulated, the mutual interdependence of the family system, makes it difficult or easy for introducing changes in the family. For example, when the partners agreed on cooking practices, participants adhered to healthy lifestyle such as dietary changes. Similar findings from other studies underline that the readiness to change were more in married couples ^30^. We also observed that when children were adamant to their preferred dietary practices, it was difficult for the family to change their dietary habits. It also demonstrates the interdependence of the family system. Secondly, the shared environment, which consists of both physical and behavioural components, are important in introducing lifestyle changes. The physical environment includes the availability, diversity, and accessibility of food and physical activity opportunities. In our study, the affordability factor is emphasised as a challenge to follow components of the SLM interventions such as adherence to recommended intake of fruits and vegetables. Similar studies from Kerala reported that food decision-making is related to cost considerations by home-makers ^31^. Therefore, a potentially valuable area of future research could be focussing on how locally available fruits and vegetables can feature in SLM messages. Participants especially women appreciated the suggestions for alternative healthier options for common recipes. Families participated in the SLM intervention narrated how they have reduced the use of oil, sugar and salt. It is largely a reflection of their ability to balance between food preferences and cost-saving. Essential items in Indian cooking such as oil and sugar, when reduced also translates to cost-saving, which is well appreciated by families.

The behavioural components of the model includes issues such as self-efficacy, self-regulation, role modelling, and feeding practices taught among family members. In our study, children were not part of several dietary changes which were made at the family level. Children are often viewed as passive receivers of food choices the families make as they do not have active role in procuring or cooking food. However, this is not the only scenario as children’s preference for taste and choices of food can control the food choices of the whole joint family ^32^. A previous qualitative study reports grandparents and other family members influence on food intake by allowing and encouraging energy dense food options for children ^33^. This highlights the need for the adaptation of SLM interventions to accommodate children and their choices.

Our study conforms to the family systems theory that places focus on the elements of individual, family and the environment together as interconnected parts of the whole ^34^. As the family system theory postulates, each element will have an influence on the desired behavioural change outcome. We found that most participants struggled with exercising; exercising for 30 to 40 minutes such as walking or yoga was a part of the SLM modification messages. Further, home makers were engaged in household chores, and therefore they did not perceive the need for additional exercise. Similarly, employed participants equated the standing or walking at work as adequate exercise. Environmental factors influencing the outcome were time , space constraints and safety. These findings are also parallel to other research conducted in Kerala, India ^35^. Such issues have also been highlighted in South Asian communities around the world ^36^, indicating a need for future studies to explore options to improve compliance to physical activity.

The PROLIFIC interventions are considered acceptable and feasible due to a variety of reasons. Firstly, the participants engaged more with the healthcare worker’s visits in the family than the written materials such as pamphlets and calendars with printed messages. Regular monitoring in the families by health workers motivated participants in the trial to adopt healthier alternatives and adhere to the SLM interventions. Secondly, participants appreciated being tracked by the ASHAs to make lifestyle changes. Similar views on being supported by health care professionals for monitoring BP and increasing physical activity are reported in other studies ^37^. Thirdly, monitoring and goal setting in families lead to better family involvement, particularly with regard to making dietary changes.

The ASHAs lifestyle messages during scheduled family visits in the PROLIFIC trial were well-received, as many participants initiated lifestyle changes. However, some of the participants reported that beginning the process of making changes to food habits or physical activity was difficult. Therefore, tailored messaging of SLM may be more useful. A broader insight from these findings is that making lifestyle changes requires external motivation in the form of active support from healthcare workers and family.

The qualitative data elicited from ASHAs were consistent with findings from other studies. Firstly, ASHAs were able to enquire and advice on smoking. However, controlling smoking and alcohol behaviours would need more support from the healthcare system. Previous studies reported that community health workers asked in detail about the initiation of smoking and advised on the importance of quitting smoking; however, they found it difficult to provide support in quitting ^38^. Another study cited that the least enjoyable part of the job was to tackle smokers, underscoring the difficulty of dealing with tobacco addiction ^39, 40^. Secondly, ASHAs reported the need for multiple visits to meet all family members. A previous study in India reported similar challenges of community workers visiting families multiple times to gather data ^39^. When all members of the family were employed, it was difficult to arrange a suitable time for the visit where the health workers could meet everyone in the family. The ASHA workers also narrated the lack of interest in meeting them on a regular basis among some of the educated family members. Additional strategies of engaging the educated group, such as utilisation of mHealth technology, may be useful in similar intervention studies. ASHAs serve as link workers ^41^ to the health care system and not as a substitute to having a consultation with a doctor or nurse. Therefore, similar intervention programmes should utilise community health workers in alignment with the needs of the population.

Training to ASHAs involved in the PROLIFIC intervention further aided participation and engagement in the study. Most ASHAs in this study were trained only in maternal and child health and the routine delivery of care for communicable diseases. Based on the feedback from the prior training sessions, ASHAs were trained to monitor BP and blood sugar during home visits in the PROLIFIC trial. This change was very well appreciated by families and was reported as one of the significant facilitators of the intervention. Additionally, the ASHAs themselves appreciated the supplementary training and skill development. Since ASHAs are not regular employees of the healthcare system, there are no options for career progression for them ^40^. The skill development enhanced their acceptability in families and acted as a motivator for them to work. Similar feedbacks of training being beneficial has been described in prior non-communicable disease programme in India ^42^. Training has also been found to be a motivating factor for community-based healthcare workers in China ^43^ and South Africa ^44^. This implies that potential screening and monitoring for cardiovascular risk in families could be offered using trained ASHAs.

The qualitative evaluation of the PROLIFIC trial helped us to introduce changes to maximise the effect of the intervention. Firstly, we increased the frequency of visits by the ASHAs to once in a month. Secondly, we trained all ASHAs in monitoring of BP and blood sugar using electronic machines. The ASHAs involved in the study monitored the BP and blood sugar of all study participants during their monthly home visits. Thirdly, ASHAs were asked to reassure the participants who have achieved optimal BP and blood sugar levels and set new lifestyle targets for those who are unable to achieve desirable levels. Fourthly, we introduced a recipe book with healthier alternatives of all regular food items in Kerala to all families in the intervention arm of PROLIFIC trial.

## Strengths and limitations

Given the qualitative and relatively small-scale nature of this study, our findings may not be applicable to other population. However, a diverse range of participants were included in our qualitative evaluation. Hence, we believe the data presented are applicable to similar other settings in low- and middle-income countries. The qualitative design used different stakeholders to gain the understanding of acceptability of the intervention. However, we do recognise the social desirability bias among participants of the intervention as they tend to highlight the positives of the intervention than negatives. The programme barriers or perceptions on remuneration from ASHAs were difficult to elicit. This is likely to be because ASHAs were paid for each house visits in the PROLIFIC study, which would have modified their responses. They would have reacted completely differently if it were conducted as part of their routine job without any incentives. Hence, the study remained descriptive with regard to elicitation of certain responses without in-depth exploration even after achieving data saturation.

## Conclusion

The findings suggest that a family based, healthcare worker led SLM intervention is desirable and feasible. However, they also highlight the importance of tailoring the lifestyle modifications suited to the participants’ needs to maximise programme adoption and utility. We identified aspects, which have been easier and more difficult to adopt and shows that interventions should be developed in consultation with participants. Further, the qualitative evaluation helped us to refine the PROLIFIC trial interventions based on the specific need of the participants.

## Data availability

### Underlying data

Figshare: PROLIFIC qualitative.zip. https://doi.org/10.6084/m9.figshare.9255758.v4 ^24^.

The file ‘PROLIFIC qualitative.zip’ contains the following underlying data:

- FGD Transcripts (de-identified transcripts from each focus group discussion).
- Participants face to face interviews (de-identified transcripts of each face-to-face interview with study participants).
- ASHA interview transcripts (de-identified transcripts of each interview with ASHAs).
- Telephonic interviews transcribed (de-identified transcripts of each telephone interview with study participants).
- Other family members interviews transcribed (de-identified transcripts of each interview with family members of study participants).

### Extended data

Figshare: PROLIFIC qualitative.zip. https://doi.org/10.6084/m9.figshare.9255758.v4 ^24^.

The file ‘Extended data.zip’ contains the following extended data:

- FGD Interview guide.docx (interview guide for focus group discussions).
- Interview Guide.docx (guides for interviews with participants, ASHAs and family members of participants).

### Reporting guidelines

Figshare: COREQ checklist for ‘Perceived facilitators and barriers of enrolment, participation and adherence to a family based structured lifestyle modification interventions in Kerala, India: A qualitative study’ https://doi.org/10.6084/m9.figshare.9255758.v4 ^21^.
