# Supplementary material for: Perceived facilitators and barriers of enrolment, participation and adherence to a family based structured lifestyle modification interventions in Kerala, India: A qualitative study
Source: Wellcome Open Res. 2019 Nov 27;4:131. Originally published 2019 Sep 6. [Version 2] doi: 10.12688/wellcomeopenres.15415.2 (PMC6896244; doi:10.12688/wellcomeopenres.15415.2)
Supplement: Peer review history

# Peer review: Reviewer response for version 2

**DOI:** 10.21956/wellcomeopenres.17072.r37175

**Published:** 2020-01-08

**Authors:** Elizabeth J. Unni

**Affiliations:** 1 Department of Social/Behavioral/Administrative Sciences, Touro College of Pharmacy, New York, NY, USA

Good with the revisions and responses to queries.

I confirm that I have read this submission and believe that I have an appropriate level of expertise to confirm that it is of an acceptable scientific standard.

# Peer review: Reviewer response for version 2

**DOI:** 10.21956/wellcomeopenres.17072.r37177

**Published:** 2020-01-07

**Authors:** Rohina Joshi

**Affiliations:** 1 Faculty of Medicine, University of New South Wales (UNSW Sydney), Sydney, Australia

The authors have revised the article in line with the suggestions. I have no further comments.

I confirm that I have read this submission and believe that I have an appropriate level of expertise to confirm that it is of an acceptable scientific standard.

# Peer review: Reviewer response for version 2

**DOI:** 10.21956/wellcomeopenres.17072.r37176

**Published:** 2019-12-05

**Authors:** Joe Varghese

**Affiliations:** 1 Department of Public Health and Tropical Medicine, Jazan University, Jizan, Saudi Arabia

I read through the revised article. I am fully satisfied with the revisions and the authors' responses.

I confirm that I have read this submission and believe that I have an appropriate level of expertise to confirm that it is of an acceptable scientific standard.

# Peer review: Reviewer response for version 1

**DOI:** 10.21956/wellcomeopenres.16855.r36410

**Published:** 2019-10-21

**Authors:** Joe Varghese

**Affiliations:** 1 Department of Public Health and Tropical Medicine, Jazan University, Jizan, Saudi Arabia

This qualitative study report is linked to SLM interventions in Prolific Trial and identifies enablers and barriers for SLM among trial participants. The study addresses an important subject and provides good details of the methodology. It also presents findings in detail. However, the following issues need further attention.

1. More justification is needed for including 50 interviews. Has 'information saturation' been considered?
2. The perspectives of the facilitators of SLM and their perspectives on barriers for participants of SLM are analysed together. In areas where facilitators reflect on the programme, issues need to be separated from their perspectives on barriers for beneficiary participation and adherence (example - section under "structured training and supporting tools" on page 11).
3. The difference between 'theme 1' and 'theme 2' is difficult to delineate. Can the second theme be more about 'adherence' rather than about “engagement and participation” (which looks the same as the first theme)? This may require a limited reanalysis of the data. The issue of/reasons for ‘adherence’ and the issue of/reasons for ‘non-adherence’ need not be the same. Therefore, your third theme will remain as it is.
4. The discussion section should reflect on some of the established theories of behavioural change, draw appropriate lessons and if possible, propose relevant hypotheses. The importance of qualitative research is its analytical generalisability which come from such reflections, without which there is a danger that this study will bring little to the body of knowledge.  

I confirm that I have read this submission and believe that I have an appropriate level of expertise to confirm that it is of an acceptable scientific standard, however I have significant reservations, as outlined above.

# Response

**Published:** 2019-11-13

**Authors:** Panniyammakal Jeemon

**Affiliations:** Sree Chitra Tirunal Institute for Medical Sciences and Technology, Trivandrum, India

**Authors’ Response to Reviewers’ Comments**

This qualitative study report is linked to SLM interventions in Prolific Trial and identifies enablers and barriers for SLM among trial participants. The study addresses an important subject and provides good details of the methodology. It also presents findings in detail. However, the following issues need further attention.

*We appreciate the comment from the reviewer and made changes in the document.*

More justification is needed for including 50 interviews. Has 'information saturation' been considered?

*We have made the following corrections to clarify the rationale for including family members. The family members were interviewed to understand any changes in lifestyle in the family due to other members participating in the intervention. The data saturation was achieved after 10 interviews.*

*The rationale for having 40 study participants is that we had a purposive sampling frame. The aim was to recruit both genders, employed and homemakers and achieve data saturation independently in each group. Initially the interviewers could not arrange face-to-face interviews with employed participants and hence had to switch to telephonic interviews.*

*To examine if there is any difference in being employed, and making life style changes the rest of the interviews were carried on and were stopped on achieving data saturation.*

The perspectives of the facilitators of SLM and their perspectives on barriers for participants of SLM are analysed together. In areas where facilitators reflect on the programme, issues need to be separated from their perspectives on barriers for beneficiary participation and adherence (example - section under "structured training and supporting tools" on page 11).

*Our objective was to describe participants’ and ASHA worker's perspectives of barriers and facilitators of enrolment, participation and adherence to a structured lifestyle modification (SLM) interventions as part of the PROLIFIC trial in Kerala, India. Therefore, the barriers and facilitators were analysed together for ‘enrolment’ ‘participation’ and ‘adherence’.*

The difference between 'theme 1' and 'theme 2' is difficult to delineate. Can the second theme be more about 'adherence' rather than about “engagement and participation” (which looks the same as the first theme)? This may require a limited reanalysis of the data. The issue of/reasons for ‘adherence’ and the issue of/reasons for ‘non-adherence’ need not be the same. Therefore, your third theme will remain as it is.

*We thank the reviewer for this comment and have re-analysed the themes. The current themes are theme 1 (Motivation for enrolment and participation) and theme 2 (Perceived explanations for engagement and adherence)*

The discussion section should reflect on some of the established theories of behavioural change, draw appropriate lessons and if possible, propose relevant hypotheses. The importance of qualitative research is its analytical generalisability which come from such reflections, without which there is a danger that this study will bring little to the body of knowledge.  

*We have added established family based theories through which the findings are discussed. The findings of the study for family member’s motivation for enrolment and participation have been examined using the double ABCX family theory **[1]**.* *The findings of perceived explanations for engagement and adherence and non-adherence themes have been compared using family model suggested by Vednathan et al **[2]** and Family systems theory. **[3]***

[1] McCubbin, H. I., & Patterson, J. M. (1983). The family stress process: The double ABCX model of adjustment and adaptation. *Marriage & Family Review*, *6*, 7–37.

[2] Vedanthan R, Bansilal S, Soto AV, Kovacic JC, Latina J, Jaslow R, Santana M, Gorga E, Kasarskis A, Hajjar R, Schadt EE, Björkegren JL, Fayad ZA, Fuster V. Family-based approaches to cardiovascular health promotion. J Am Coll Cardiol . 2016;67(14):1725–1737.

[3] C.B. Broderick. Understanding FAMILY PROCESS: Basics of Family Systems Theory. Sage Publications, Thousand Oaks, CA (1993)

# Peer review: Reviewer response for version 1

**DOI:** 10.21956/wellcomeopenres.16855.r36482

**Published:** 2019-10-14

**Authors:** Rohina Joshi

**Affiliations:** 1 Faculty of Medicine, University of New South Wales (UNSW Sydney), Sydney, Australia

**Review: **This well written qualitative study aims to understand the perceptions of patients and providers about the enrolment, participation and adherence to a lifestyle management study nested in a trial (PROLIFIC). Please find below my comments which may help strengthen the paper.

**Introduction:**

- “Based on previous systematic reviews and studies among Indians living in high-income countries, barriers for lifestyle changes are predominantly related to lack of knowledge and misunderstanding of CVD risk factors ^10^” – please add other references (systematic reviews and studies), or if it is one study then change to “based on a previous systematic review”

**Methods**

- While the study method is stated as a qualitative study, the methods described read like part of a process evaluation of a trial. Please clarify if this is a stand alone qual or part of a larger process evaluation.
- Also, was a theoretical framework such as REAIM used to underpin the methods and analyse the findings?
- “It was conducted as part of the ongoing evaluation of acceptability in terms of ‘reach’ and ‘fidelity’ of the PROLIFIC trial interventions.” How was ‘Reach’ evaluated? And how was fidelity captured? There is no mention of reach and fidelity in the results.
- Some details about the intervention would help the reader understand the study further. E.g. what was done at each visit? How were the interventions tailored? Was this is paper based or used digital health?
- The term NPHW and ASHA is used interchangeably, I would suggest that once the NPHW is identified as the ASHA, the term ASHA should be used in the rest of the paper. 

**Results & Discussion**

- The majority of participants were women, please explain how that influenced the results? Among the non-responders, were the majority males?
- “Training to NPHWs involved in the PROLIFIC intervention further aided participation and engagement in the study.” – was the training of ASHAs evaluated, if so, how? Please clarify. 
- “Most ASHAs were trained only in maternal and child health and the routine delivery of care for communicable diseases ^36^”. – Please explain this statement, as NPCDCS program is being rolled out in India. Also, the reference given is incorrect.
- Re. motivation of ASHAs, the discussion states that training is a key factor in motivating ASHAs due to the new skill set learnt and respect earned by family members and the communities. Training is a motivating factor, evidence (including from India) shows that both financial and non-financial factors are important. Please explain if the ASHAs given an incentive as part of PROLIFIC?
- “This implies that potential screening and monitoring for cardiovascular risk in families could be offered using trained NPHW”. There are several studies which now show that when trained, community health workers like ASHAs can screen, monitor and follow up individuals at high risk of CVD. The national NCD program trains ASHAs to screen (based on previous history and symptoms) and follow up individuals with NCDs. The issue is that ASHAs not being formal employees of the health system are overworked and underpaid. Hence if the intervention is scaled up, how would it truly empower ASHAs? The authors have raised this issue in the limitations section, could they further explain how could this intervention be implemented at scale?

I confirm that I have read this submission and believe that I have an appropriate level of expertise to confirm that it is of an acceptable scientific standard, however I have significant reservations, as outlined above.

# Response

**Published:** 2019-11-13

**Authors:** Panniyammakal Jeemon

**Affiliations:** Sree Chitra Tirunal Institute for Medical Sciences and Technology, Trivandrum, India

**Authors’ Response to Reviewers’ Comments:**

Review: This well written qualitative study aims to understand the perceptions of patients and providers about the enrolment, participation and adherence to a lifestyle management study nested in a trial (PROLIFIC). Please find below my comments which may help strengthen the paper.

Introduction:

“Based on previous systematic reviews and studies among Indians living in high-income countries, barriers for lifestyle changes are predominantly related to lack of knowledge and misunderstanding of CVD risk factors10” – please add other references (systematic reviews and studies), or if it is one study then change to “based on a previous systematic review”

*We appreciate this comment. We have added the relevant references.*

Methods

While the study method is stated as a qualitative study, the methods described read like part of a process evaluation of a trial. Please clarify if this is a stand alone qual or part of a larger process evaluation.

*We would like to clarify that this was a standalone qualitative study for evaluation of acceptability, barriers and facilitators of PROLIFIC interventions during the course of the study.*

Also, was a theoretical framework such as REAIM used to underpin the methods and analyse the findings?

*We would like to clarify that no theoretical framework was used for the analysis. The data analysis followed a thematic analysis as described by Braun and Clarke.*

“It was conducted as part of the ongoing evaluation of acceptability in terms of ‘reach’ and ‘fidelity’ of the PROLIFIC trial interventions.” How was ‘Reach’ evaluated? And how was fidelity captured? There is no mention of reach and fidelity in the results.

*We would like to clarify that the sentence was phrased incorrectly. We have changed the sentence to “It was conducted as part of the ongoing evaluation, in terms of acceptability, ‘reach’ and ‘fidelity’ of the PROLIFIC trial interventions”*

*The evaluation of ‘reach and fidelity’ was done using* *quantitative tools including feedback in diary, monthly chart review of set goals etc. which will be published as a separate paper.*

Some details about the intervention would help the reader understand the study further. E.g. what was done at each visit? How were the interventions tailored? Was this is paper based or used digital health?

*More details of the intervention component are already published in our methods paper. Jeemon P, Harikrishnan S, Sanjay G, Sivasubramonian S, Lekha TR, Padmanabhan S, Tandon N, Prabhakaran D. A PROgramme of Lifestyle Intervention in Families for Cardiovascular risk reduction (PROLIFIC Study): design and rationale of a family based randomized controlled trial in individuals with family history of premature coronary heart disease. BMC Public Health. 2017 Jan 5;17(1):10. doi:10.1186/s12889-016-3928-6.*

*In this qualitative study we have focused more on the acceptability, barriers and facilitators to the delivery of interventions by ASHA workers at the family level. We have cited the methods paper while describing the methodology of PROLIFIC study.*

The term NPHW and ASHA is used interchangeably, I would suggest that once the NPHW is identified as the ASHA, the term ASHA should be used in the rest of the paper.

*We appreciate this suggestion and the terms have been replaced with ASHAs.*

Results & Discussion            

The majority of participants were women, please explain how that influenced the results? Among the non-responders, were the majority males?

*The majority of the trial participants were also women. We recruited high risk families with one index case of premature coronary heart disease. Since the incidence of CHD is higher in men, we had relatively higher number of women participants as family members. However, the non-responders were more males for the telephonic interviews.*

“Training to NPHWs involved in the PROLIFIC intervention further aided participation and engagement in the study.” – was the training of ASHAs evaluated, if so, how? Please clarify.

*The ASHA training was not formally evaluated. However, we had conducted FGDs with ASHAs and they perceived that the training component increased their confidence to interact with the family members and deliver the interventions at the family level. Further, training in BP measurement and blood glucose measurement helped them to engage the participants in the interventions.*

“Most ASHAs were trained only in maternal and child health and the routine delivery of care for communicable diseases36”. – Please explain this statement, as NPCDCS program is being rolled out in India. Also, the reference given is incorrect.

*We thank the reviewer for pointing the error in reference. We have changed the reference.*

*Most ASHAs recruited in this programme had no formal training given through NPCDCS for measuring blood pressure or blood sugar. The junior public health nurses (JPHN) under NPCDCS take the blood pressure during the house visits. We had conducted one full day hands-on training in measurement of BP and blood glucose for ASHAs. Even after one full day training, and taking a minimum of 3-4 supervised measurements by each ASHA, some of them were not able to do it independently. Additional, training was given to them by our project staff.*

Re. motivation of ASHAs, the discussion states that training is a key factor in motivating ASHAs due to the new skill set learnt and respect earned by family members and the communities. Training is a motivating factor, evidence (including from India) shows that both financial and non-financial factors are important. Please explain if the ASHAs given an incentive as part of PROLIFIC?

*We appreciate this query. ASHAs were paid an honorarium as part of PROLIFIC study to cover their travel expenses as recommended by the NHM mission office. However, this was not mentioned by ASHAs during interviews. It was however highlighted as limitation of the qualitative study.*

“This implies that potential screening and monitoring for cardiovascular risk in families could be offered using trained NPHW”. There are several studies which now show that when trained, community health workers like ASHAs can screen, monitor and follow up individuals at high risk of CVD. The national NCD program trains ASHAs to screen (based on previous history and symptoms) and follow up individuals with NCDs. The issue is that ASHAs not being formal employees of the health system are overworked and underpaid. Hence if the intervention is scaled up, how would it truly empower ASHAs? The authors have raised this issue in the limitations section, could they further explain how could this intervention be implemented at scale?

*We agree that ASHAs are involved in multiple programmes and they are underpaid. However, most of the ASHA consider it as part of social service. Our initiative is a targeted approach to high risk families. It is easy to link each incident case of CHD and their family to a health worker during the discharge time itself (ASHA or any other NPHW) and therefore it is potentially scalable. In PROLIFIC trial we evaluate the effectiveness of this approach in CVD risk reduction. In order to scale-it up at the state or national level, we may have to invest more on the training component of ASHAs. We are also aware of the research findings from other studies in India that clearly state that ASHAs have the potential to deliver a broad range of services, if supported by the health system appropriately. We will be able to offer more comments on scale-up after the effectiveness, cost-effectiveness, reach and fidelity analyses.*

*Since we paid honorarium to ASHAs as part of PROLIFIC study, the scaled up programme would need the similar model for functioning.*

# Peer review: Reviewer response for version 1

**DOI:** 10.21956/wellcomeopenres.16855.r36409

**Published:** 2019-09-10

**Authors:** Elizabeth J. Unni

**Affiliations:** 1 Department of Social/Behavioral/Administrative Sciences, Touro College of Pharmacy, New York, NY, USA

This is a good study and written well. However, it needs few more edits and revisions to make it more sound to a reader.

**Introduction **- The authors talk about the incidence and prevalence of CVD and IHD in India. However, since the study is done in Kerala, a few statistics about these diseases in Kerala would benefit the reader.

**Methods **- Data collection - It says "three independent female researchers conducted all the semi structured interviews..." Will you please explain why the gender of the researchers is important? Were most of the interviewed people also female? Does it have a cultural naunce? Please explain. Otherwise, if not relevant, please remove it from the text.

**Data Analysis** - From the introduction towards the end, there are two aims for the study; acceptability and facilitators and barriers. But in the data analysis section, it is only talking about facilitators and barriers. Please confirm.

 The analysis says "the themes were compared against the study objectives to ensure that those significantly contributed towards the research question...". This process is not clear. Please clarify.

**Ethical Approvals** - Were there any incentives given to the interview and FGD participants? Please explain.

**Results **- There were 40 in depth interviews. That is a lot of interviews. Please explain how you reached this number? 

 Figure 1 is a repetition of data from Page 4. Please remove the figure. Instead, please add a table that summarizes the interview and FGD questions. It is hard to relate the answers when the questions are not provided. 

 Though 40 of your interviews were with study participants and 10 with family members, the study results are grouped. It says "family's experience of participating in the trial..". Please clarify. If the goal was to obtain family's experience, there has to be a rationale for having 40 study participants and only 10 family members. 

 Results section and Table 2 also talks only about the facilitators and barriers to participation. The results of the aim of understanding the "acceptability" of the program is not clear. Please clarify.

**Discussion** - The authors talk about the acceptability of the program and has given reasons (page 12 towards the end). But that is not clear from the results section. Is "motivation to participate" theme considered as "acceptability"? Because they participated in this study, it cannot be extrapolated to think that the program is acceptable to them. That is where the conclusions drawn from the study are not fitting well with the study results. The results demonstrate the facilitators and barriers in participating in the study. But it does not talk about the acceptability of the program. Also, the authors talk about the issue of remuneration. Without additional remuneration, will ASHAs continue this work? What about the study participants? Were they paid to be a part of this study? Is the program sustainable and feasible?

**Conclusion - **Says "...SLM intervention is acceptable and feasible". Please provide more data to support this conclusion.

I confirm that I have read this submission and believe that I have an appropriate level of expertise to confirm that it is of an acceptable scientific standard, however I have significant reservations, as outlined above.

# Response

**Published:** 2019-11-13

**Authors:** Panniyammakal Jeemon

**Affiliations:** Sree Chitra Tirunal Institute for Medical Sciences and Technology, Trivandrum, India

**Authors’ Response to Reviewers’ Comments:**

*Firstly, the authors express their appreciation to the three reviewers and the editor. We believe that their vital comments and suggestions have contributed substantially to improve the presentation of our study, as well as its overall quality and the manuscript. Following, we offer point-by-point replies to the issues and points the reviewers addressed regarding the original manuscript.*

This is a good study and written well. However, it needs few more edits and revisions to make it more sound to a reader. *We are grateful for the reviewer’s precise, detailed, and constructive comments and suggestions, after which we have revised the manuscript text, tables, figures, and reference list as described below and in the revised manuscript.*

**Introduction **- The authors talk about the incidence and prevalence of CVD and IHD in India. However, since the study is done in Kerala, a few statistics about these diseases in Kerala would benefit the reader.

*We have added the burden of CVD and IHD in Kerala.*

**Methods **- Data collection - It says "three independent female researchers conducted all the semi structured interviews..." Will you please explain why the gender of the researchers is important? Were most of the interviewed people also female? Does it have a cultural naunce? Please explain. Otherwise, if not relevant, please remove it from the text.

*We would like to clarify that there is no cultural nuance or importance for the gender of the interviewers. We have updated the sentences as follows “*three independent researchers conducted all the semi structured interviews..."

**Data Analysis** - From the introduction towards the end, there are two aims for the study; acceptability and facilitators and barriers. But in the data analysis section, it is only talking about facilitators and barriers. Please confirm.

*We would like to clarify that the results presented are for barriers and facilitators for participation and adherence to lifestyle management.*

The analysis says "the themes were compared against the study objectives to ensure that those significantly contributed towards the research question...". This process is not clear. Please clarify.

*We appreciate the comment and the sentence have been changed to "the transcripts were analysed and themes were identified so as to address the research question” to ensure clarity.*

**Ethical Approvals** - Were there any incentives given to the interview and FGD participants? Please explain.

  

*For the ASHA workers (female community health workers) travel allowance was paid for attending the FGD. In-depth interviews with family members or ASHAs were not incentivized.*

**Results **- There were 40 in depth interviews. That is a lot of interviews. Please explain how you reached this number? 

*We had a purposive sampling frame for trial participants. The aim was to recruit both genders, employed and homemakers. Initially the interviewers could not arrange face-to-face interviews with employed participants and hence had to switch to telephonic interviews. To examine if there is any difference in being employed, and making life style changes the rest of the interviews were conducted and stopped on achieving data saturation.*

Figure 1 is a repetition of data from Page 4. Please remove the figure. Instead, please add a table that summarizes the interview and FGD questions. It is hard to relate the answers when the questions are not provided. 

*We have added the interview and FGD interview guides. However, we are retaining the Figure 1 for better visual representation of the themes.*

Though 40 of your interviews were with study participants and 10 with family members, the study results are grouped. It says "family's experience of participating in the trial..". Please clarify. If the goal was to obtain family's experience, there has to be a rationale for having 40 study participants and only 10 family members. 

*Kindly note that, the PROLIFIC study is a family based intervention trial. The trial participants are also family members. We have a minimum of two participants in the trial from each family.* *The non-participant family members in the PROLIFIC trial were also interviewed to understand any changes in lifestyle in the family beyond the trial participants. The data saturation was achieved after 10 interviews. Together, the data provide the family perspective on participating in the PROLIFIC trial.*

Results section and Table 2 also talks only about the facilitators and barriers to participation. The results of the aim of understanding the “acceptability” of the program is not clear. Please clarify.

*We would like to clarify that the results presented are for barriers and facilitators for participation and adherence to lifestyle management. However, acceptability can be viewed as a precondition for adherence. Without acceptability, adherence does not occur. Therefore, facilitators for adherence can be viewed as acceptable factors. (Sekhon et al **[1]**, Blenkinsopp et al **[2]**).*

Discussion – The authors talk about the acceptability of the program and has given reasons (page 12 towards the end). But that is not clear from the results section. Is “motivation to participate” theme considered as “acceptability”? Because they participated in this study, it cannot be extrapolated to think that the program is acceptable to them. That is where the conclusions drawn from the study are not fitting well with the study results.

*We would like to clarify that the “motivation to participate” theme is not considered as “acceptability”. This theme relates to participant’s initial decisions in joining the program. The acceptability, referred to was the acceptance of intervention delivered by the ASHA workers. Monitoring by ASHA workers by itself was perceived as a facilitator for lifestyle changes and therefore the trial interventions were perceived as acceptable.*

The results demonstrate the facilitators and barriers in participating in the study. But it does not talk about the acceptability of the program. Also, the authors talk about the issue of remuneration. Without additional remuneration, will ASHAs continue this work? What about the study participants? Were they paid to be a part of this study? Is the program sustainable and feasible?

*We thank the reviewer for the questions on remuneration. This factor is highlighted as a necessary factor to take into account for the sustainability of such program. The study participants were not paid. We have not enquired into the sustainability of the program in this study. The feasibility of the program for this qualitative study was looking at the ease of intervention delivery by the health workers.*

Conclusion - Says "...SLM intervention is acceptable and feasible". Please provide more data to support this conclusion.

*We have explained the acceptability in the response 10. Feasibility in this study refers to the factors contributing ASHAs in delivering the intervention, which have been addressed in the study.*

[1] Sekhon, M., Cartwright, M. & Francis, J.J. Acceptability of healthcare interventions: an overview of reviews and development of a theoretical framework. *BMC Health Serv Res* **17, **88 (2017) doi:10.1186/s12913-017-2031-8

[2] Blenkinsopp A, Hassey A. Effectiveness and acceptability of community pharmacy‐based interventions in type 2 diabetes: a critical review of intervention design, pharmacist and patient perspectives. Int J Pharm Pract. 2005;13(4):231–40.
